# Identifying the Preferred Subset of Enzymatic Profiles in Nonlinear Kinetic Metabolic Models via Multiobjective Global Optimization and Pareto Filters

**DOI:** 10.1371/journal.pone.0043487

**Published:** 2012-09-20

**Authors:** Carlos Pozo, Gonzalo Guillén-Gosálbez, Albert Sorribas, Laureano Jiménez

**Affiliations:** 1 Departament d'Enginyeria Química (EQ), Escola Tècnica Superior d'Enginyeria Química (ETSEQ), Universitat Rovira i Virgili (URV), Tarragona, Spain; 2 Departament de Ciències Mèdiques Bàsiques, Institut de Recerca Biomèdica de Lleida (IRBLLEIDA), Universitat de Lleida, Lleida, Spain; Center for Genomic Regulation, Spain

## Abstract

Optimization models in metabolic engineering and systems biology focus typically on optimizing a unique criterion, usually the synthesis rate of a metabolite of interest or the rate of growth. Connectivity and non-linear regulatory effects, however, make it necessary to consider multiple objectives in order to identify useful strategies that balance out different metabolic issues. This is a fundamental aspect, as optimization of maximum yield in a given condition may involve unrealistic values in other key processes. Due to the difficulties associated with detailed non-linear models, analysis using stoichiometric descriptions and linear optimization methods have become rather popular in systems biology. However, despite being useful, these approaches fail in capturing the intrinsic nonlinear nature of the underlying metabolic systems and the regulatory signals involved. Targeting more complex biological systems requires the application of global optimization methods to non-linear representations. In this work we address the multi-objective global optimization of metabolic networks that are described by a special class of models based on the power-law formalism: the generalized mass action (GMA) representation. Our goal is to develop global optimization methods capable of efficiently dealing with several biological criteria simultaneously. In order to overcome the numerical difficulties of dealing with multiple criteria in the optimization, we propose a heuristic approach based on the epsilon constraint method that reduces the computational burden of generating a set of Pareto optimal alternatives, each achieving a unique combination of objectives values. To facilitate the post-optimal analysis of these solutions and narrow down their number prior to being tested in the laboratory, we explore the use of Pareto filters that identify the preferred subset of enzymatic profiles. We demonstrate the usefulness of our approach by means of a case study that optimizes the ethanol production in the fermentation of *Saccharomyces cerevisiae*.

## Introduction

Genetic manipulation of microorganisms for obtaining improved strains involves expensive and time consuming experiments that have typically relied on trial-and-error mutagenesis and selection of promising variants. Nowadays, mathematical models of cell metabolism and gene regulation circuits have become reliable enough for metabolic engineering applications [1]–[3]. These models can be coupled with optimization algorithms in order to identify the most promising genetic manipulations leading to an enhanced phenotype in a given microorganism. This approach requires defining a suitable objective function, for instance the maximum yield or flux of interest. Optimization is then performed by considering the model equations describing the microorganisms' metabolism and a set of constraints relevant for cell viability [4]–[8]. This method provides, a sound theoretical basis for experimentalists on the best strategies for manipulating the biological system, either by changing enzyme levels through genetic manipulations or by altering environmental conditions [9].

The selection of an appropriate mathematical model is a crucial step towards success in this field. Two main strategies can be followed at this stage. On the one hand, one can choose mathematical simplicity and a genome-wide scope. In this context, flux balance analysis (FBA) provides an appropriate solution (for a full list of abbreviations used in this paper, please refer to Nomenclature S1). This method makes use of stoichiometric models to represent the metabolic networks, which gives rise to mixed-integer linear formulations (MILP) that are easy to solve with standard techniques [10]. This MILP approach, however, fails at capturing the regulatory loops existing in metabolic networks [11]. On the other hand, one can choose a kinetic detailed description, which necessarily will be limited to relatively few pathways at a time. Detailed kinetic models can deal with all kind of regulatory signals and reaction mechanisms, but involve nonlinear equations (e.g., Michaelis-Menten, Hill or power-law, etc.) required to appropriately represent the reaction rates as a function of the involved metabolite concentrations. These nonlinearities give rise to nonconvexities which in turn lead to the potential existence of multiple local optima (i.e., multimodality). This may prevent standard algorithms from identifying the global optimum, as they can get trapped in local wells during the search. Global optimization strategies overcome this limitation, guaranteeing convergence to the global optimum within a desired tolerance. It should be emphasized that global optimization is of paramount importance in these theoretical biological studies since misidentifying a local optimum as the global one may lead to spurious conclusions [12], [13].

For S-Systems models, a particular class of power-law models, Voit [4] proposed a reformulation strategy based on a logarithmic transformation that brings the model to an LP/MILP form, making it possible to apply standard optimization methods that ensure global optimality. This reformulation cannot be applied to other non-linear models, such as GMA models or detailed kinetic models. These last models must be tackled though using global optimization methods. One such method for GMA models based on an outer approximation algorithm was proposed by Polisetty et al. [8]. Guillén-Gosálbez and Sorribas [12] presented further developments using an outer approximation-based algorithm [14] and related advanced strategies [12], [15] to globally optimize GMA models. These methods have been recently extended further to deal with detailed kinetic models through a mathematical reformulation framework termed recasting that converts them into GMA models [13].

Biotechnology studies typically seek optimizing a single flux in the metabolic network as unique criterion. In practice, however, there are other criteria of interest for experimentalists, such as minimizing the number of enzymatic changes, metabolic concentration of intermediates [16] or transient times [17]. Despite the importance of such additional criteria, the majority of works in metabolic engineering are based on single-objective formulations. Although some of these functional criteria can be treated as constraints ensuring cell viability, they should be treated as additional objectives [18]. This would eventually allow for the identification of solutions in which cell viability is further improved at the expense of marginal reductions in other objectives such as growth.

**Figure 1:**
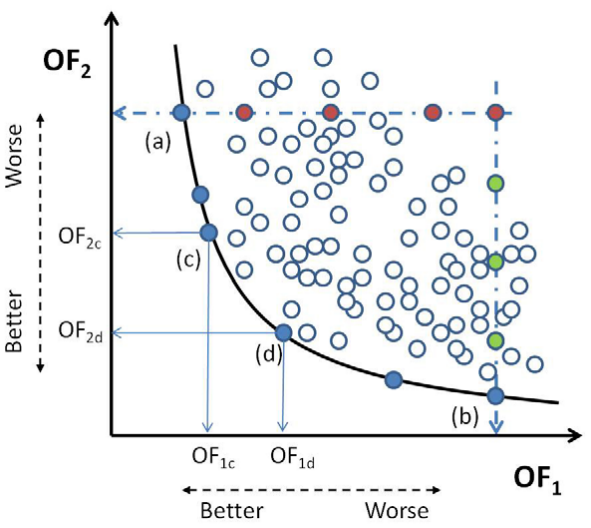
Generic Pareto front. Full blue points indicate members of the pareto set. Point (a) is the optimum for objective function 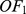 for a given value of 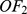 (red points). Point (b) minimizes 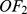 for another value of 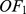 (compared to green points). For a member of the Pareto set, say (c), any attempt to improve a goal involves worsening the other, point (d) for comparison. Empty blue points are other possible solutions that are worse than those in the Pareto set.

The importance of multiobjective optimization in metabolic studies has been pointed out by several authors [19]–[21]. Technically, the solution of a multiobjective optimization (MOO) problem is given by a set of points known as the Pareto set. All these solutions feature the property that it is not possible to find another one that improves any of them in one objective without worsening at least one of the others (see Figure 1). Because of the presence of continuous variables, optimization problems arising in metabolic engineering may have an infinite number of Pareto-optimal solutions. Clearly, testing all these alternatives in the laboratory would be prohibitive in terms of time and resources. Multi-criteria decision-making (MCDM) can be of great help at this stage to rank and/or screen alternatives, ruling out the less promising and keeping the best. Unfortunately, the complexity of both, MOO and MCDM, increases with the number of objectives. In practice, the visualization and analysis of the Pareto set becomes highly difficult in problems with more than three objectives. The need for advanced methods to support these tasks in biochemical systems has already been acknowledged [21], [22].

Several approaches have been proposed for identifying a subset of Pareto solutions of special interest for decision-makers. For instance, Branke et al. [23] and later Deb [24] suggested either to specify the extreme pair-wise trade-off information about objectives or to attach relative weights to them, in order to concentrate the search in a particular region of the Pareto set. Branke and Deb [25] proposed a projection-based method to obtain a biased distribution of Pareto solutions. Farina and Amato [26] introduced a more restrictive dominance concept that produces less number of Pareto solutions. Branke et al. [27] introduced a method for obtaining those Pareto solutions with a significantly different slope (i.e., “knee” solutions). Deb and Gupta [28] focused on identifying robust (i.e. less sensitive to parameter changes) solutions. The concept of Pareto filter was also employed by several authors for eliminating non-Pareto or locally optimal Pareto solutions [29]–[33].

MOO and MCDM have been extensively studied in the context of a wide variety of engineering problems (for instance, refer to [34]). In contrast, their application to metabolic engineering has been quite scarce [35]. In this work, we address the MOO of metabolic networks. Our study assumes a GMA model of the target metabolic network where all model parameters are known. These include the stoichiometric coefficients of the reactions involved in the production/consumption of each internal metabolite; and the parameters of the power-law formalism that model the kinetics of each reaction at the basal state. Then, we will seek the optimization of a given flux assuming two important complementary objectives: (i) We assume that any increment in gene expression is a limiting factor for the cell as it involves an important metabolic burden; (ii) We also consider that an excessive increment in intermediate concentrations compromises cell viability. These two criteria will be used as complementary objectives that should be minimized when possible.

Under these conditions, we aim to develop a systematic framework to (i) calculate the Pareto front of the kinetic metabolic model in this multi-objective problem and (ii) identify from it a small enough set of the most promising changes in enzyme activity to be tested in the laboratory. In other words, the goal of this analysis is to determine a set containing the preferred enzymatic profiles that optimize the synthesis rate of a metabolite at minimum cost (minimum number of changes in these activities, i.e. minimum change in gene expression) and minimum increase in the concentration of intermediate metabolites.

Note that there are two main difficulties associated with the identification of such set. First, we need to solve a high dimensional non-convex multiobjective optimization problem in which several criteria must be simultaneously minimized. This problem is challenging not only because of the high number of objectives, but also due to the existence of non-convexities. Second, even if a sufficiently large number of Pareto solutions can be identified, there is still the issue of analyzing and interpreting them, in order to keep the most promising for further evaluation in the laboratory. Deb and Saxena [36] reviewed the main difficulties associated with the calculation and analysis of the Pareto solutions of MOO problems with large number of objectives, like those arising in metabolic engineering. As will be shown later in the paper, our systematic approach allows overcoming some of these difficulties.

In particular, our strategy relies on the combined use of multiobjective global optimization and Pareto filters, which are both applied to metabolic networks described using the GMA formalism. The method presented builds upon our global optimization framework for single-objective models of metabolic networks [14], [37], which is adequately modified herein to handle multiple objectives. This method is based on an outer approximation algorithm that decomposes the target problem into a master MILP and a slave NLP, which respectively provide lower bounds (LB) and upper bounds (UB) on the global optimum. These bounds tend to approach as iterations proceed until a given tolerance is satisfied.

Note that our methodology shares some common features with that presented by [35] for S-Systems models. However, while the former strategy ends with the generation of the Pareto optimal front, ours goes one step beyond by suggesting a subset of preferred alternatives that are identified using Pareto filters. Hence, this work presents advances in two main fronts: (i) the generation of Pareto optimal solutions for multiobjective GMA models, and (ii) the identification of the most promising alternatives using systematic filters.

The capabilities of the proposed methodology are illustrated in the optimization of the fermentation of *Saccharomyces cerevisiae* considering 14 objectives. This process has been already studied in the past by several authors. For instance, Sendín et al. [35] used an ad-hoc model of this metabolic pathway to address by means of different MOO methods a 6-objective MOO problem considering the ethanol synthesis rate and the concentration of 5 dependent metabolites. Most of the approaches compared therein show some limitations, as they either rely on local solvers (this is the case of weighted sum, attainment goal and NBI) or employ stochastic optimization methods (MOEA) that are unable to guarantee convergence to the global optimum in a finite number of iterations, which may result in a spurious Pareto front. The other method studied in that work (MIOM) requires the transformation of the original model into an S-Systems representation, which is something unnecessary when relying directly on GMA models. Furthermore, we address here a more complex problem that accounts for 14 objectives (the fold-change in 8 different enzyme activities, expressed as the absolute value of the natural logarithm of the enzyme activity fold-change; the concentration of 5 dependent metabolites; and the ethanol synthesis rate). This represents a significant advance compared to traditional biotechnological approaches that maximize the ethanol yield and impose biological constraints for maintaining metabolites and enzymes levels around their basal state so as to preserve cell homeostasis [9].

## Results

In order to illustrate the capabilities of our approach we solved a case study that optimizes the ethanol production in the fermentation of *Saccharomyces cerevisiae*. For this, steps 2 and 4 of the algorithm proposed (refer to the Methods Section for further details) were coded in GAMS 23.2.0, while the normalization step 3 was implemented off-line using Microsoft Excel. Numerical experiments were performed on an Intel 1.2 GHz. The GMA model (Step 1) was retrieved from [8]. The reader is referred to this paper for further technical details. Bounds on metabolite concentrations and changes in enzyme activities were the same as those reported in [14].

Note that we assume that the GMA model is given. If this was not the case, a previous step would be necessary to construct such a model from dynamic profiles using parameter estimation methods. We should note also that the modeling software GAMS is a versatile tool that allows implementation of all the framework's steps, offering standard coding capabilities and interfacing with powerful optimization solvers.

### Obtention of the Pareto set

**Figure 2:**
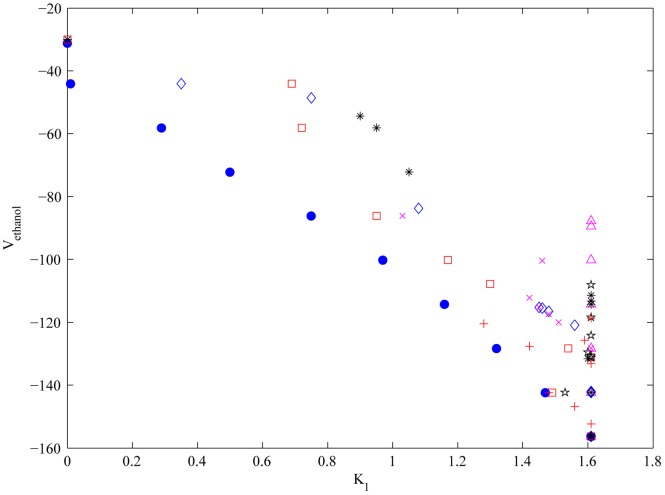
Pareto curve (blue circles) of the bi-criteria problem considering 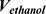
 **and** 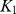 **(Hexose transporters).** The other points represent projections of the same variables obtained during other bi-criteria optimization problems: 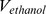-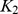 (red squares), 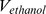-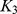 (magenta triangles), 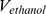-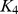 (black stars), 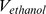-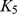 (blue diamonds), 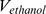-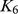 (red plus signs), 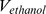-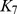 (magenta cross signs) and 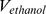-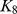 (black asterisks). Fold-Change factors correspond to: 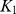: Hexose transporters, 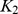: Glucokinase/Hexokinase, 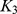: Phosphofructokinase, 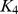: Trehalose 6-phosphate syntase complex (+Glycogen production), 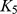: Glyceraldehyde-3-phosphate dehydrogenase, 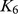: GOL (Glycerol production), 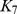: Pyruvate kynase, 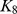: ATPase.

The MOO problem was solved using the epsilon constraint method, which was enhanced through a heuristic procedure based on generating solutions for all possible bi-criteria subproblems. We defined 10 epsilon parameters for each objective, which gave rise to 910 single iterations (note that the same number of objectives and epsilon intervals would lead to more than 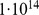 instances using the traditional epsilon constraint approach). The outer approximation-based algorithm [14], [37] was then employed to solve these instances to global optimality. CPLEX 11.2.1 was used as MILP solver for the lower bounding master problem, and CONOPT 3.14 s for the slave NLPs. All the sub-problems of the algorithm were solved to global optimality within a tolerance of 0.2%, which is the same tolerance that we used in [14] for the analogous single objective problem. A set of Pareto optimal solutions was finally obtained through the above commented procedure. Figure 2 shows the 2D Pareto set for the maximization of 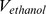 vs minimization of hexose transporters (i.e. 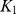) changes. As observed, as we increase the value of 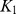 (recall that we are representing 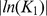), the ethanol synthesis rate increases. In the same Figure, we have also projected the points resulting from the other bi-criteria optimizations, that is, in Figure 2 we have included also the points obtained from the optimization of 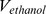-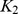, 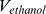-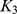, …, 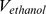-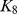. As observed, while there is a clear tendency in the points coming from one bi-criteria optimization, the same is not true when we consider the remaining solutions generated by the other bi-criteria results. Hence, while we can “easily” analyze the trade-off between two single objectives, it is difficult to perform the same analysis when several criteria come into play.

**Figure 3:**
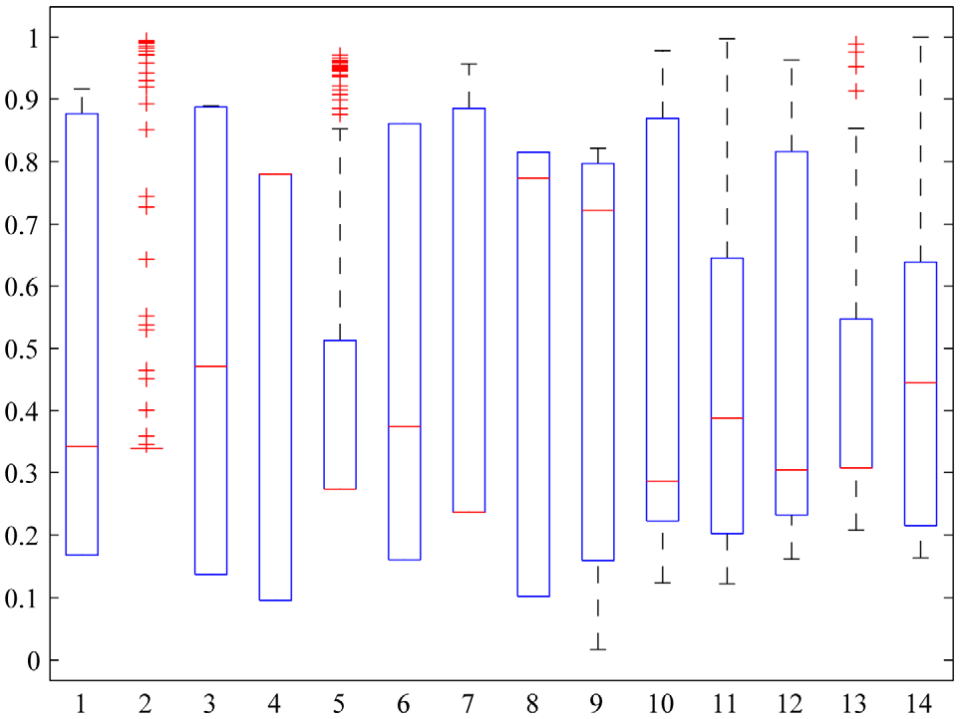
Box plot for the normalized Pareto set. In the bottom axis the fourteen objectives are represented. Objectives 1–8 correspond to 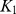
–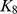 (see legend in Figure 2), objective 9 is indeed 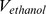 whereas the remaining 5 objectives represent 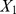
–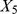. 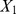: Internal glucose, 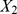: Glucose-6-phosphate, 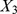: Fructose-1,6-diphosphate, 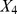: Phosphoenolpyruvate, 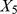: Adenosine triphosphate.

The Pareto set was next normalized (see the Section “Normalization of the Pareto optimal solutions” in Methods) assuming a normal distribution for all objectives. We further assumed that the mean and standard deviation are the same as those of the samples (i.e., the solutions generated with the epsilon constraint method). Note that this brings the data to the [0,1] range. Figure 3 shows the box plot associated with the normalized Pareto solutions. As seen, objective 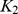 shows a very small variability (the 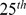 and 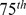 percentiles correspond to the same value, around 0.34, as the median). This implies in turn that it is easy to obtain a good (i.e. small) value for this objective. The same happens in the case of objectives 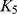, 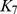, 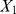, 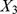 and 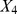, for which the median and 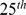 percentile are also rather close, indicating that the solutions are concentrated around their minimum values. On the contrary, most solutions are allocated at high (i.e., poor) values of objectives 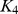, 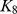 and 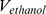, while very few are close to their minimum values.

### Selection of preferred subset of solutions

The Smart filter was applied next in order to remove indistinguishable solutions from the pool. The application of this algorithm has also the effect of providing a more uniform spread of points. Note that choosing larger values of tolerance 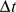 will allow discarding more solutions from the pool, but this may come at the expense of loosing valuable solutions (i.e., promising enzymatic profiles). To illustrate this, we performed the calculations for two different values of 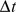. In particular, selecting a 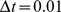 allowed to reduce the size of the Pareto set from 910 to 611 solutions, whereas only 321 solutions were retained for a 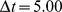. We found that using a 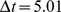 resulted in an excessive loss of information in this case study, and hence, kept the results obtained with a 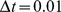.

We next resort to the second type of Pareto filter: the order of efficiency filter. We started by imposing a 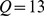 (i.e., 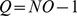), and searched for nondominate solutions in any of the 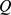-elements subsets of objectives. This narrowed down the number of Pareto solutions from 611 to 214 alternatives. The procedure was repeated for decreasing values of 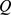 until an empty set of solutions was identified, which occurred for a value of 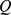 = 10. In particular, 14 solutions were found to be efficient of order 12, while only 1 solution was efficient of order 11.

**Figure 4:**
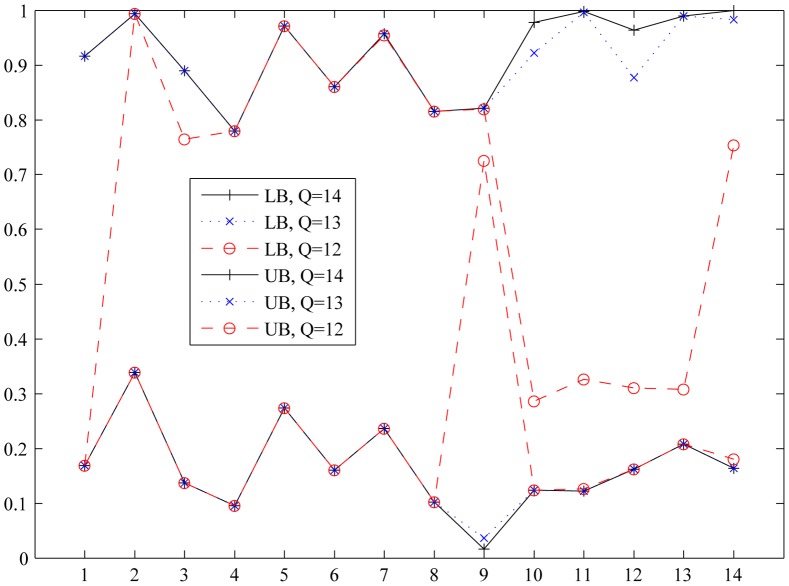
Lower and upper bounds for objectives among the values attained by the set of Pareto solutions of order 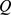
 **.** In particular, 611 solutions are efficient of order 14 (i.e., these are indeed the solutions obtained after applying the Smart filter); 214 solutions are efficient of order 13; and 14 solutions are efficient of order 12. Objectives are ordered as in Figure 3. See legends in Figure 2 and 3.

Figure 4 shows the minimum and maximum objective values among those solutions retained for a given 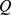. This plot provides valuable insight on how much quality is lost as we decrease efficiency order. The closer the lower bound curve of a set of solutions is to the lower bound curve of the original set, the better is the quality of the set, as this implies that such set contains solutions with objective function values close to the best possible performance that can be attained in each criterion.

Particularly, the lower and upper limits of the 214 solutions efficient of order 13 are quite close to the bounds corresponding to the 611 solutions of the Pareto set obtained using the Smart filter, showing a small decrease (about 2%) in the ethanol synthesis rate with respect to the maximum possible value. There are 14 solutions efficient of order 12 with a curve rather close in most objectives to that of the 611 original solutions. In this set, however, the ethanol synthesis rate drops by an additional 69%, which is consistent with the trend observed in Figure 3. We should clarify that it is possible to artificially add in the final pool of solutions any other alternative for further consideration, with special interest on those with good performance in one criterion and poor in the others that are not efficient of order 12.

Remarkably, the only solution efficient of order 11 (which is not included in Figure 4) is not the closest to the utopia point, that is, it is not the one with the minimum Euclidean distance to the utopia point, which is a common criterion for selecting a single final candidate from a Pareto set.

Table 1 shows the values obtained for the 14 objectives in the solutions efficient of order 12. It can be seen that some of the solutions are very close to the ethanol production rate of the basal solution (i.e., solution with the 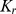 values fixed to one), which turns out to be 30.11 mM min^−1^ [8]. The best solution comprising only three changes in enzyme activity achieves a ethanol production rate of 37.68 mM min^−1^ and involves a 2.3 fold increase in 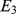 (which corresponds to a 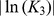 = 0.84), and about a 5 fold increase in 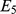 and 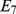. A ethanol production rate of 42.88 mM min^−1^ can be achieved by changing four enzymes. This leads to a 42% increase over the basal production rate. In this case, 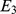 must be modified by a factor of 3.5, 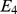 5 times, 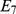 2.1 times, and 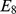 1.7 times approximately. Further increases in ethanol production would require manipulating a larger set of enzymes. Single objective optimization focusing on maximizing the ethanol production would obtain better yields, but would entail higher (costly) enzyme changes and probably higher metabolic concentrations that would compromise the cell viability.

**Table 1:** 14 solutions efficient of order 12 in decreasing order of 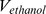.

*K*_1_	*K*_2_	*K*_3_	*K*_4_	*K*_5_	*K*_6_	*K*_7_	*K*_8_	*V*_ethanol_	*X*_1_	*X*_2_	*X*_3_	*X*_4_	*X*_5_
0.00	0.00	0.86	1.61	1.16	0.00	1.16	1.61	43.27	0.06	0.26	3.27	<0.01	0.34
0.00	0.00	1.26	1.61	0.00	0.00	0.75	0.56	42.88	0.05	0.26	16.93	<0.01	0.94
0.00	0.00	1.14	1.61	1.52	0.00	0.15	0.82	41.95	0.05	0.26	1.49	0.01	0.70
0.00	0.00	0.97	1.61	0.00	1.31	0.39	1.07	38.36	0.05	0.26	16.59	0.01	0.44
0.00	0.00	0.84	0.00	1.61	0.00	1.59	0.00	37.68	0.04	0.47	0.91	<0.01	1.48
0.00	0.00	0.59	1.61	0.00	0.00	0.81	0.20	35.83	0.04	0.55	12.15	<0.01	1.14
0.00	1.61	0.56	1.61	0.00	1.57	0.25	1.58	34.97	0.01	0.29	16.53	0.01	0.22
0.00	1.00	1.17	1.61	1.61	1.61	0.05	0.28	34.43	0.01	0.26	0.91	0.01	0.74
0.00	1.18	0.00	0.00	1.48	0.00	0.00	1.22	33.53	0.01	0.64	1.25	0.01	0.37
0.00	0.00	0.00	0.00	0.00	1.30	0.53	1.35	32.20	0.04	0.58	13.66	<0.01	0.29
0.00	0.00	0.00	0.00	0.00	1.29	0.55	1.30	32.17	0.04	0.59	13.50	<0.01	0.31
0.00	0.00	0.57	0.00	1.61	1.61	0.00	0.86	31.46	0.05	0.36	0.91	0.01	0.37
0.00	1.61	0.45	1.61	0.00	1.29	0.16	0.02	30.54	<0.01	0.60	9.69	0.01	0.98
0.00	0.00	0.44	1.61	0.00	1.61	0.44	0.00	30.24	0.04	0.61	9.54	<0.01	0.98

Recall that columns labeled as *K*_r_ represent indeed 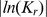. Enzyme 1: Hexose transporters, enzyme 2: Glucokinase/Hexokinase, enzyme 3: Phosphofructokinase, enzyme 4: Trehalose 6-phosphate syntase complex (+Glycogen production), enzyme 5: Glyceraldehyde-3-phosphate dehydrogenase, enzyme 6: GOL (Glycerol production),enzyme 7: Pyruvate kynase, enzyme 8: ATPase, metabolite 1: Internal glucose, metabolite 2: Glucose-6-phosphate, metabolite 3: Fructose-1,6-diphosphate, metabolite 4: Phosphoenolpyruvate, metabolite 5: Adenosine triphosphate.

## Discussion

In this paper, we have introduced a systematic framework for the multiobjective deterministic global optimization of metabolic networks modeled through the GMA formalism. The proposed strategy integrates the epsilon constraint method, deterministic global optimization tools, and a set of Pareto filters that narrow down the final number of candidate solutions to be tested in the laboratory. The method presented does not rely on any visualization procedure, being therefore suitable for problems with a large number of objectives. The capabilities of the proposed approach were illustrated by means of a benchmark problem that addressed the optimization of the ethanol synthesis rate in *Saccharomyces cerevisiae*.

Biological objectives, such as the concentration of intermediate metabolites and the enzymatic changes were considered in addition to the ethanol synthesis rate. By selecting the auxiliary problems of the epsilon constraint method in a smart way, we could reduce the computational burden considerably. Furthermore, the Pareto filters allowed reducing the number of promising alternatives significantly from 910 to 14 (i.e., 98% reduction), illustrating the usefulness of the approach in the post optimal analysis of the candidate solutions. In different test problems, the outer approximation algorithm integrated in our systematic framework efficiently solved problems with up to 30 independent metabolites and 60 reactions in short CPU times (i.e., few minutes). Hence, we expect the method to scale up smoothly when tackling more complex models, even though we have yet to explore its limits. Note, however, that genome-wide scale problems are still beyond the capabilities of current deterministic global optimization methods. First, there is a lack of kinetic data to build realistic genome scale models. Second, assuming the existence of a detailed enough kinetic model, there is still the issue of solving it to global optimality in short CPU time. For these reasons, genome scale models are usually solve via FBA, despite the known limitations of this method. Nevertheless, we think that advances in deterministic global optimization theory and software applications will pave the way for more efficient algorithms leading to significant CPU savings, which will make it possible to tackle complex genome scale kinetic models.

In summary, our approach allows for the global optimization of metabolic networks on different objectives simultaneously. The method presented reduces the computational burden associated with the generation of solutions, and facilitates the post-optimal analysis of these alternatives by systematically identifying the best ones (i.e., more balanced) for subsequent experiments in the laboratory. Hence, our method is particularly suited for problems of moderate size. Larger kinetic models could be tackled with stochastic methods, but even if they are the method of choice, it will be still possible to use the Pareto filters introduced in our work. However, we will not have any information on the quality of the solution found. Finally, for genome-wide scale models, FBA might be the method of choice, despite having some limitations already discussed in the literature.

## Methods

**Figure 5:**
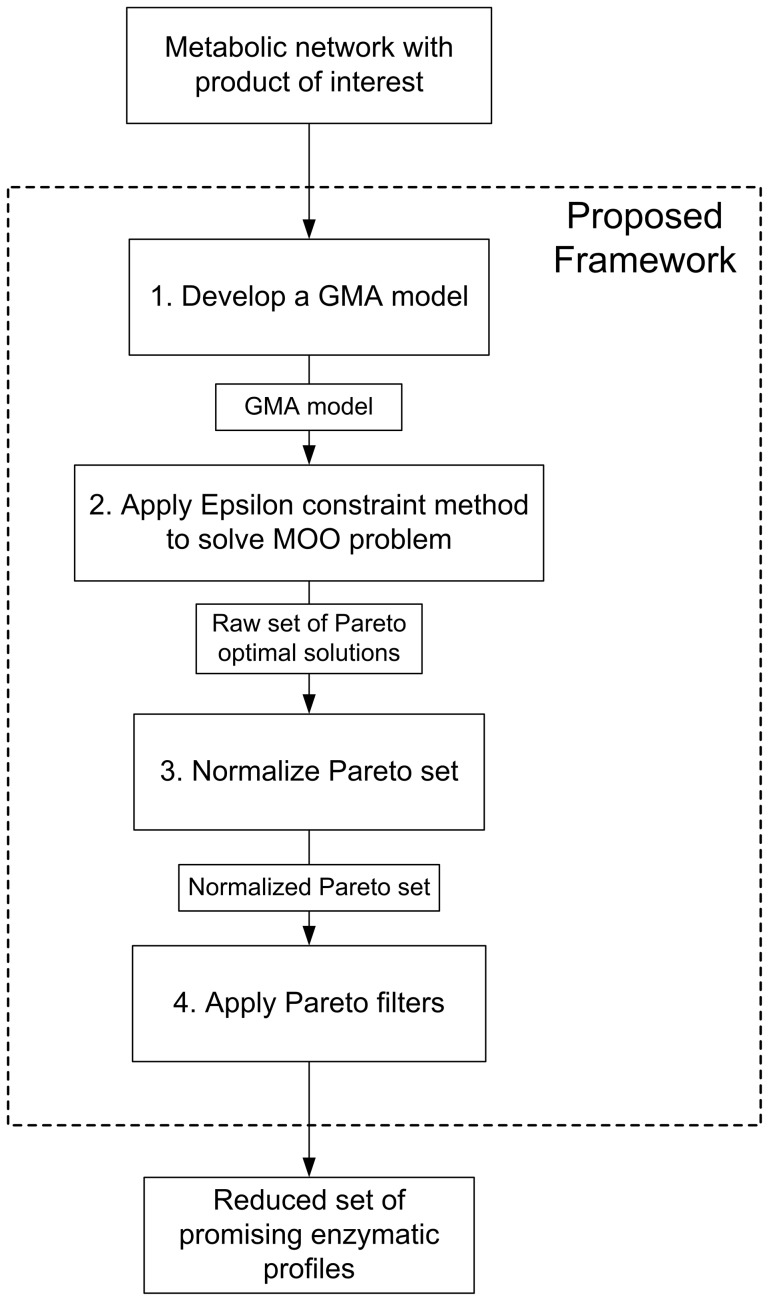
Proposed algorithm for the multiobjective global optimization of metabolic networks. This method allows not only to generate a Pareto set, but also to systematically select the most promising subset of enzymatic profiles embedded therein.

Our systematic framework comprises the following steps (see Figure 5):

1. Model building and parameter estimation (optional): construct a GMA model for the targeted metabolic network.
2. Global optimization of the GMA model on several biological criteria.
3. Normalization of the solutions obtained in step 2.
4. Application of Pareto filters to identify the preferred subset of alternatives.

The sections that follow describe in detail each of these steps.

### Mathematical model: GMA representation

The optimization of the metabolic network is posed in mathematical terms as a multiobjective NLP (i.e., moNLP) that embeds GMA equations. Note that there are different possible ways to obtain this GMA model. Particularly, we can follow a top-down approach, that is, find the parameters of a GMA model that make it consistent with dynamic data by solving a parameter estimation problem. On the contrary, we might be interested in following a bottom-up strategy and acquire the GMA model of interest from the literature. In what follows, we describe briefly the GMA formalism before presenting the details of the moNLP.

We assume that the concentration 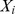 of every metabolite 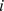 present in a metabolic network varies with time 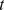 as a result of the action of 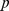 flows:
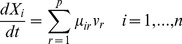
(1)The stoichiometric coefficient, 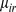, appearing in Eq. 1 is an integer parameter accounting for the number of molecules of metabolite 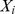 that are involved in the process 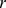. It is positive when the reaction 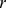 produces metabolite 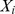 and negative when 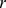 consumes 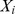. Note that not all the 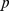 processes in the metabolic network are directly involved in the production of every single metabolite 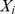, which implies that some parameters 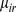 are zero (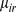 = 0) for some particular combinations of 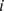 and 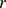. The velocity at which process 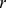 occurs, is represented using the so-called power-law formalism [38]–[40] as in Eq. 2.
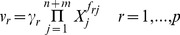
(2)Here, 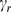 is a parameter denoting the basal state activity of the enzyme governing process 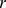, whereas 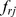 is the kinetic order of metabolite 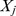 in process 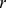. This representation accounts for the 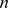 internal dependent and 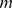 external (i.e., independent) metabolites. At this point, the concentration of the external metabolites will be considered fixed. Thus, the term 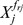 behaves as a variable for 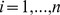 and as a parameter for 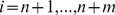. By combining Eq. 2 and Eq. 1, we obtain a GMA model (Eq. 3).
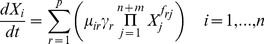
(3)To model the effect of genetic manipulations performed on the strain, we introduce an auxiliary continuous variable, 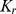 that accounts for the fold-change over the basal state enzymatic level 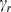 as follows:
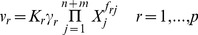
(4)Recall that, in Eq. 4, the product 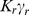 denotes the actual enzyme activity. Hence, the values of 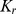 in the optimal solution will dictate the modification to be performed in the strain: 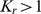 indicates overexpression of enzyme 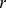, 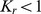 denotes its downregulation, and a value of 1 means that enzyme 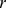 is not manipulated. Furthermore, bounds 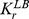 and 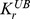 are imposed on this variables as stated in Eq. 5.
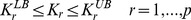
(5)Similarly, metabolite concentrations are allowed to change within given bounds (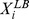 and 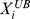, respectively):
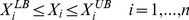
(6)


Since we are interested in solving the steady state, the time dependence can be dropped from the formulation:
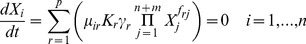
(7)


For demonstrative purposes, we assume that the main objective is to maximize the synthesis rate of a desired product. This rate is calculated by summing up the velocities of those processes contributing to its synthesis, as illustrated in Eq. 8.
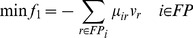
(8)Here, 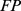 is the set of metabolites 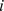 that are regarded as final products and 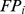 is the set of processes 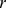 contributing to the synthesis of metabolite 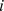 (i.e., those processes for which 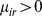). Note that, for simplicity, we have posed the problem as a minimization one by reversing the sign of the objective function.

Two additional criteria are appended to the objective function. The first is the minimization of the metabolites concentrations, proposed as an optimality principle for metabolic networks [16]. Genetic manipulation of many genes at once may be costly and technically difficult. To take this into account, the model seeks to minimize the individual changes in enzyme activities. The resulting MOO problem that embeds the GMA equations can be expressed in compact form as follows:
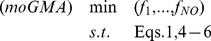
(9)Thus, model 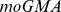 seeks the appropriate changes in enzyme activities (continuous variable 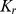) that maximize simultaneously the synthesis rate of the desired product and minimize the concentration of the metabolites and changes in enzyme activities. Objective 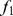 represents the synthesis rate targeted, while 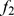 to 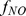 denote the metabolites concentrations 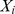 and individual changes in enzyme activities. To quantify deviations in enzyme activities from the basal state, we use the absolute value of the natural logarithm of the fold-change in enzyme activities. The enzyme activities calculated by the model can be later implemented in the real system by tuning the expressions of the corresponding genes.

The optimization problem takes the form of a nonconvex NLP, in which multiple local optima may exist. We employ global optimization techniques to ensure global optimality within a desired tolerance.

### Multiobjective global optimization of metabolic networks described by a GMA model

In general, the Pareto set of a GMA model may be nonconvex due to the nonlinear kinetic equations. Different MOO algorithms could be used to calculate this set (i.e., NBI [41], NNC [42]). We use herein the epsilon-constraint method because unlike other methods, such as the weighted sum one, it can identify points located in the nonconvex part of the Pareto set. Note that this property is also shared by the more complex NBI and NNC methods, which also offer the appealing property of providing a uniform spread of Pareto points. However, this limitation of the epsilon constraint is alleviated by coupling it with a Smart filter (refer to Section “Smart filter” in Methods). We should clarify, however, that our global optimization approach could work with other deterministic MOO algorithms, such as the NBI or NNC.

In the epsilon constraint technique, one objective is regarded as main objective, while the rest are transferred to auxiliary constraints that impose upper bounds 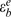 on their values:
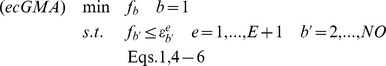
(10)


The 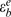 values appearing in the auxiliary constraints are commonly obtained as follows:

1. Solve problem 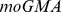 for each individual objective separately.
2. Store the best (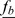) and worst (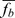) values obtained in step 1 for each objective. These values are the limits within which the auxiliary epsilon parameters must fall (i.e., 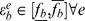).
3. Split the epsilon interval into 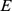 subintervals to generate parameters 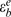 (i.e.,. 
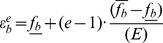
).

Note that step 1 provides the so-called anchor points, that is, the extreme solutions of the Pareto frontier.

In the traditional epsilon constraint approach, problem 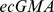 is solved for all possible combinations of 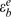, which leads to a total of 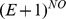 instances. The complexity of this approach grows exponentially with the number of objectives. As an example, for 3 objectives and 4 sub-intervals, we have 125 iterations; for 4 objectives and the same number of sub-intervals, we have 625, and for 5 objectives and identical number of sub-intervals, we have 3125 iterations.

Here, we follow a heuristic approach for generating Pareto solutions that consists of solving a set of bi-criteria problems corresponding to all possible combinations of any two objectives. This strategy presents some advantageous features. First, the Pareto points generated in the two-dimensional space are also Pareto optimal in higher dimensional spaces [34], and hence in the original NO-dimensional space. Second, this approach requires solving 
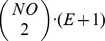
 single-objective models, rather than 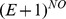, which dramatically reduces the computational effort. For instance, it would reduce the number of iterations required in the previous example from 125 to 15, from 625 to 30 and from 3125 to 60, respectively.

The epsilon constraint method transforms the MOO problem into a set of single-objective problems. This is very convenient, since it makes it possible to apply our global optimization methods devised for single-objective GMA models [14], [37] to multiobjective problems. In particular, in this work we use the outer-approximation-based algorithm we developed in [14], which was inspired by the works of Polisetty et al. [8] and Bergamini et al. [43].

Following this approach, the original problem (i.e., 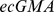 in this case) is divided into two subproblems at two different hierarchical levels. A master problem consisting of a linear relaxation of 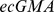 is solved in the upper level to predict a LB on the global optimum. A slave problem based on the original model is then solved locally in the lower level using the solution of the master problem as starting point in order to predict an UB. The solutions computed during the first iteration are used to tighten the relaxation of the master problem, which will produce better LBs in subsequent iterations. The algorithm proceeds in this manner until the optimality gap (OG, defined as the relative difference between the UB and the LB) is reduced below a given tolerance.

The most important step of the outer approximation is the construction of the master MILP problem. This MILP is built by applying an exponential transformation that brings the model into a canonical form that can be relaxed in a straightforward manner using piecewise linear approximations and supporting hyper-planes. For the sake of brevity, technical details about this procedure are omitted herein. The interested reader is referred to the original works by Pozo et al. [14], [37] for further details.

### Normalization of the Pareto optimal solutions

A normalization procedure is applied to the Pareto set of solutions in order to bring them to the same scale and units, so they become readily comparable. A plethora of alternative methodologies are available for this purpose. One of the main drawbacks of normalization methods is that they tend to concentrate the points in some regions of the feasible domain.

In a recent work, Cloquell et al. [44] presented a normalization methodology previously proposed in another work [45] that aims at overcoming this limitation. According to this strategy, the normalized value of a given solution 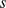 is calculated as follows:
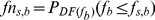
(11)Where 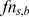 is the normalized value associated with the non-normalized value 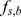, and 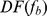 is the probability distribution function of the objective variable 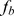. The form of this distribution is assumed beforehand, with the normal distribution being the common choice.

### Pareto filters

The previous steps provide as output a set of normalized Pareto points. As mentioned previously, an infinite number of such points may exist for problems involving continuous variables. Testing all of them in the laboratory would be highly expensive and time consuming. Hence, a method is required for screening and ranking then, narrowing down their total number. We explore the application of two different Pareto filters. A Smart filter [46] is applied first to remove indistinguishable alternatives from the pool. A second filter based on the order of efficiency of the Pareto solutions [47] is then employed to identify solutions that are well-balanced, that is, they show “good” performance simultaneously in all of the objectives.

#### Smart filter

**Figure 6:**
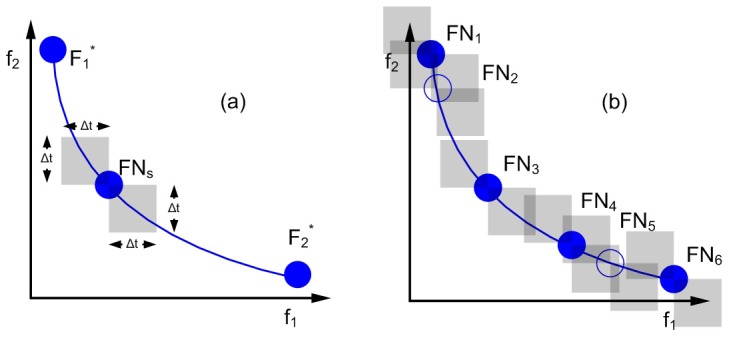
Illustration of the smart Pareto filter. a) Indistinguishability region. b) Algorithm performance example.

Two arbitrary solutions that are rather close in the objective space might be equally appealing for decision-makers, despite representing completely different experimental manipulations. If any of these is preferred over the other because of differences in any of the required changes, this differentiating feature should then be regarded as an additional objective [46]. A possible way to reduce the size of the Pareto set is to eliminate solutions which are within a given tolerance in the objectives space, that is, solutions which entail insignificant differences compared to others. Figure 6 illustrates the underlying idea behind this filter. As seen in Figure 6a, a region is defined around each normalized solution 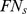. Any other solution 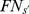 falling inside this region is said to be indistinguishable from 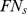, and automatically removed from the pool. Consider for instance the example presented in Figure 6b where a small set of solutions is presented. We start by comparing solution 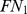 with the rest, and then removing those contained inside the shaded region defined around the reference point. After comparing all the points, we pick the next candidate solution and repeat the procedure again. In this particular example, solution 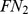 is found within the specified tolerance of 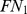 and 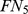 is within the region defined by 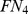.

To this end, we use the following algorithm, which is based on that presented by Mattson et al. [46]:

Let 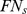 be one of the 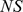 normalized solutions of the normalized Pareto set (i.e., 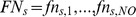) obtained through steps 2 and 3 of the solution approach, and let 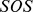 be the set containing all these solutions. The application of the filter comprises the following steps.

1. Define tolerance 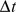, a set of rejected solutions 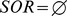, a set of candidate solutions 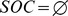 and start iteration counters 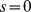 and 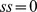.
2. While 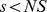,

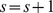

  - If 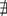 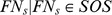, return to 2.a. Else:
  - While 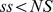,

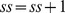

    - If 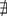 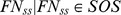, return to 2.c.i. Else:
    - If 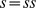, return to 2.c.i. Else, if 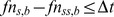 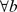, let 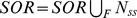 and 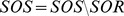.
  - End while
  - Restart iteration counter ss = 0.
3. End while
4. Make 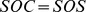

We should clarify that this algorithm is a special case of the one proposed by Mattson et al. [46], in which the original 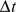 and 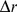 are assumed to be equal to 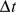. Furthermore, note that the value of this control parameter is the same in all of the objectives, since the Pareto points are normalized prior to the application of the filter.

This filter is particularly useful when coupled with the epsilon constraint method, as it alleviates its tendency to concentrate points in given regions of the Pareto front, thus giving rise to a more uniform spread of points.

#### Order of efficiency filter

The filter described above allows reducing the number of Pareto solutions. Further reductions can be attained by applying the concept of order of efficiency, as introduced by Das [47]. A solution is said to be efficient of order 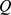 if it is not dominated by any other solution in any of the possible 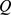-elements subsets of objectives. In mathematical terms, a solution 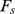 is said to be efficient of order 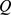, if and only if, 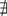 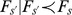 for any subset of objectives of cardinality 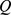. In this definition, we consider that a solution 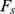 dominates 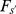 (i.e., 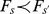) if and only if, 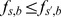 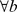 with at least one 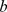 in which 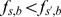.

**Figure 7:**
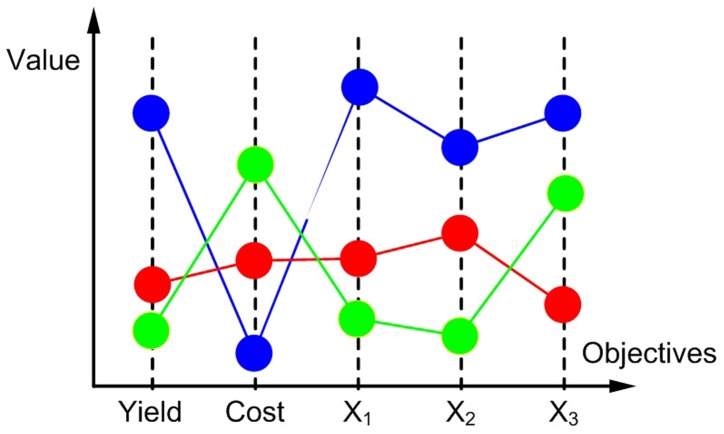
Illustrative example for the Pareto order of efficiency concept. Blue solution is efficient of order 5, whereas red solution is efficient of order 4 and green solution is efficient of order 3.

Figure 7 provides an illustrative example of the concept of Pareto efficiency of order 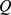. Consider we have a MOO problem with 5 biotechnological criteria: final product yield, aggregated cost of changing the enzyme activities via gene expression, and concentration of 3 different metabolites (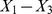). Assume that the values of 3 different solutions (blue, read and green) have already been normalized as described previously, so that the minimum value of each of the 5 objectives represents their individual optima. As seen, the three solutions plotted are Pareto optimal since none of them can improve any of the others simultaneously in all of the objectives. At this point, one can start eliminating solutions which are not efficient of order 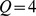 by identifying sets of 4 objectives in which a given solution is dominated. For instance, the blue solution is dominated by the green and red ones in the set 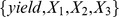. On the other hand, the red solution is not efficient of order 3, since it is in turn dominated by the green one in 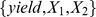. Hence, the green solution is the only one that is efficient of order 3, while none of them is efficient of order 2 (i.e., the green solution is dominated by the red one in 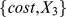).

According to the definitions previously exposed, if a solution is efficient of order 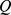, it is also efficient of order 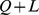 with 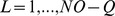 (see [47] for proofs). Note that the concept of efficiency of order 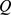 is stronger than the Pareto optimality condition [47], and can thus be used to discern between efficient alternatives. Furthermore, this concept avoids the use of any arbitrary “criterion of merit” or visualization technique, making it suitable for high-dimensionality problems [47].

We propose to apply this filter for searching efficient solutions of order 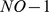, and then repeat the process recursively for successively inferior orders of efficiency until either an empty set is found or the number of solutions retained is sufficiently small. As pointed out by Das [47], solutions with lower order of efficiency are expected to be well-balanced. This is because solutions behaving well in some objectives but poorly in others are expected to be dominated at least in the subsets including the latter criteria [47].

## Supporting Information

Nomenclature S1 **The abbreviations and variables used in this paper.**
(PDF)
