# Supplementary material for: Identifying the Preferred Subset of Enzymatic Profiles in Nonlinear Kinetic Metabolic Models via Multiobjective Global Optimization and Pareto Filters
Source: PLoS One. 2012 Sep 20;7(9):e43487. doi: 10.1371/journal.pone.0043487 (PMC3447875; doi:10.1371/journal.pone.0043487)
Supplement: Nomenclature S1 — The abbreviations and variables used in this paper. (PDF) [file pone.0043487.s001.pdf]

Nomenclature S1

Abbreviations

FBA	Flux Balance Analysis
GMA	Generalized Mass Action
LB	Lower Bound
LP	Linear Programming problem
MCDM	Multi-criteria decision-making
MILP	Mixed-Integer Linear Programming problem
MOO	Multiobjective Optimization
moNLP	Multiobjective Non-Linear Programming problem
NLP	Non-Linear Programming problem
OG	Optimality gap
UB	Upper Bound

Indices

e	subintervals in the epsilon-constraint method
i	dependent (internal) metabolites
j	metabolites (dependent + independent)
r	flows
b	objectives

Sets/Subsets

FP	set of metabolites i that are regarded as final products
FP_i	set of processes r contributing to the synthesis of metabolite i
SOC	set of candidate solutions s
SOR	set of rejected solutions s
SOS	set of normalized Pareto solutions s

Parameters

NO	total number of objectives b
E	total number of subintervals e in the epsilon-constraint method
f_{rj}	kinetic order of metabolite j in process r
K_r^{LB}	Lower bound on fold-change in the activity of enzyme r
K_r^{UB}	Upper bound on fold-change in the activity of enzyme r
m	total number of independent (external) metabolites j
n	total number of dependent (internal) metabolites i
NS	total numbr of normalized Pareto solutions s
p	total number of flows r
Q	order of efficiency of a Pareto optimal solution s
X_i^{LB}	lower bound on the concentration of metabolite i
X_i^{UB}	upper bound on the concentration of metabolite i
Δt	tolerance control parameter for Smart Pareto filter
ϵ_b^e	lower bound of subinterval e of objective b in epsilon-constraint method
γ_r	basal state activity of enzyme governing process r
μ_{ir}	stoichiometric coefficient of metabolite i in reation r

Variables

f_b	value of individual objective b
$f_{s,b}$	value of individual objective b in solution s
$fn_{s,b}$	normalized value of individual objective b in solution s
FN_s	vector containing the normalized values of all objectives in solution s
K_r	fold-change over the basal activity of enzyme r
X_i	concentration of metabolite i
t	time
v_r	velocity of process r
